# Early life social conditions and adverse experiences are associated with childhood BMI and perceived overeating

**DOI:** 10.1111/ijpo.13179

**Published:** 2024-10-08

**Authors:** Anna Bartoskova Polcrova, Gabriela Ksinan Jiskrova, Martin Bobak, Hynek Pikhart, Jana Klánová, Albert J. Ksinan

**Affiliations:** ^1^ RECETOX, Faculty of Science Masaryk University Brno Czech Republic; ^2^ Department of Epidemiology and Public Health University College London London UK

**Keywords:** ACEs, BMI, childhood obesity, overeating, socioeconomic deprivation

## Abstract

**Background:**

Early life socioeconomic disadvantage and adverse experiences may lead to overeating, which is in turn associated with increased body mass index (BMI). However, recent evidence indicated that the association between childhood BMI and overeating might be bidirectional. This bidirectionality prompts the need for further investigation of early life predictors of BMI in childhood.

**Objectives:**

To longitudinally assess the directionality of the association between childhood BMI and perceived overeating and to investigate their antecedent early life predictors.

**Methods:**

The sample included data from 5151 children from the ELSPAC study, collected between 18 months and 11 years of child age. The outcomes were child BMI and mother‐reported overeating, assessed at the age of 3, 5, 7 and 11 years. Predictors included maternal BMI, maternal education, single parenthood, financial difficulties and adverse childhood experiences (ACEs) reported by parents and paediatricians. The random intercept cross‐lagged panel model was applied.

**Results:**

The mean child's BMI at age 3 was 15.59 kg/m^2^ and increased to 17.86 kg/m^2^ at age 11. The percentage of parent‐reported overeating increased in the following period, from about 12% at age 3 to 17% at age 11. The results showed temporal stability in perceived overeating and BMI, with a bidirectional relationship strengthening over time. The child's BMI was associated with maternal BMI. Maternal BMI was positively associated with child‐perceived overeating, but a stronger effect was found for ACEs. ACEs mediated the impact of maternal education, financial difficulties and single parenthood on overeating.

**Conclusions:**

We observed stable bidirectional associations between BMI and perceived overeating. The results indicated two main pathways: one linked to maternal BMI and early childhood BMI increase followed by perceived overeating and the second associated with ACEs mediating the effect of early childhood social factors on perceived overeating, leading to gradual BMI gain.

## 1 INTRODUCTION

Obesity stands out as a significant and pressing public health issue. Along with the escalating global prevalence of obesity amongst adults, a noticeable rise is also observed amongst children and adolescents. Based on the World Obesity Atlas,^1^ the 2020 overall prevalence of obesity amongst children and adolescents aged 5–19 years was 19% in boys and 12% in girls.^1^ The progression of obesity is a continuous process involving a complex system of genetic, environmental and behavioural factors that influence an individual's susceptibility to excess adiposity.^2^ This trajectory begins in prenatal stages and continues through infancy, childhood, adolescence and adulthood, and all those phases of life represent potential targets for public health interventions. The relationships between factors can also develop and change dynamically over the life course.^3^

In 2016, the Commission on Ending Childhood Obesity presented a set of recommendations to address childhood obesity, incorporating preconception and pregnancy care, promoting healthy food intake and physical activity in early childhood and school‐age children, and weight management.^4^ Consistently to these recommendations, previous interventions focused mainly on inappropriate eating behaviours or insufficient physical activity in children, mothers and families.^5^ However, this approach might be insufficient in light of recent knowledge and recommended practices for individuals living with obesity. The Clinical Practice Guideline for the Evaluation and Treatment of Children and Adolescents with Obesity conducted by the American Academy of Pediatrics postulated that obesity should be considered a chronic disease determined by an interconnected system of factors at national, community, family and individual levels.^6^ The multifactorial origin of paediatric obesity establishes a need for a broader perception of risk factors. In particular, it is necessary to focus on factors beyond the scope of individual behaviours.

Determinants beyond the level of individual factors can be collectively referred to as upstream determinants.^2^, ^7^, ^8^ Upstream determinants originate from various societal, economic, environmental and structural elements that influence health outcomes and behaviours.^7^, ^8^ The social inequalities present in these upstream determinants play a pivotal role in driving disparities in childhood obesity. Children from disadvantaged environments characterized by low parental education,^9^ financial difficulties^10^ or single parenting^11^ are particularly vulnerable to obesity development. This increased vulnerability may occur due to the lack of resources in families suffering from economic deprivation. Besides, disadvantaged social environments are more likely to result in negative life occasions, such as adverse childhood experiences (ACE), including physical, emotional, or sexual abuse, as well as parental mental health illnesses or family dysfunction.^12^, ^13^ ACEs have been previously determined as risk factors for obesity in childhood,^8^, ^14^, ^15^ but at the same time, their effect persists into later years, increasing the risk of obesity in adulthood.^15^

Upstream determinants might also affect an individual's ability to regulate food intake.^16^ Socioeconomic disadvantage as well as psychological distress may lead to overeating, in this context typically referred to as emotional overeating.^17^ Based on the existing literature, the common assumption has been that overeating leads to increased energy intake, followed by weight gain.^18^ However, recent evidence indicated that the association between childhood obesity and overeating might be, in fact, bidirectional. A study conducted by Derks et al. in 2018 on a sample of Dutch children showed that higher body mass index (BMI) at preschool predicted overeating at a later age with a stronger association than the previously hypothesized reverse direction.^19^ Similarly, another study conducted on Hispanic children in 2020 provided evidence for the bidirectional longitudinal relationship between BMI and overeating.^20^

This bidirectionality of the association prompts the need for further investigation of the pathways of effects of previously identified early life predictors of increased BMI in childhood. It is thus important to evaluate whether distinct upstream determinants independently predict overeating and BMI, and whether the effect of such upstream determinants on childhood BMI can be potentially explained by the longitudinal development of overeating as a continuous accompanying phenomenon. Reflecting this notion, the current study contains two main aims, the first being to longitudinally assess the directionality of the association between BMI and perceived overeating during childhood and the second to investigate the effects of early life upstream determinants on longitudinal trends in both perceived overeating and BMI. We hypothesize that there will be a significant positive bidirectional relationship between BMI and perceived overeating throughout childhood. Further, we hypothesize that ACEs will be positively associated with increased levels of BMI. We also hypothesize that ACEs will be positively associated with increased levels of perceived overeating. Next, we hypothesize that the effect of social determinants on BMI will be mediated by ACEs. Similarly, we hypothesize that the effect of social determinants on perceived overeating will be mediated by ACEs.

## 2 METHODS

### 2.1 Study population and study sample

The data originated from the Czech arm of the European Longitudinal Study of Pregnancy and Childhood (ELSPAC); a prospective longitudinal birth cohort study conducted across six countries. Initially, pregnant women residing in the Brno region or Znojmo district, expected to deliver between 1 March 1991 and 30 June 1992 were included in the study. The baseline dataset included information from 5151 mothers and their newborns.^21^ Following the initial enrollment, parents provided responses to questionnaires regarding health, lifestyle, dietary patterns, demographic details, psychosocial aspects and environmental exposures. This information was collected when the children were 6 and 18 months old, and subsequently, data collection occurred when the children reached ages 3, 5, 7, 11, 15, 18 and 19 years. Starting at 11, children began contributing self‐reports alongside reports from their parents and other sources at the same age, including medical records provided by children's paediatricians.

### 2.2 Measures

#### 2.2.1 BMI

Children's height and weight were obtained at four timepoints around the age of 3, 5, 7 and 11 years from the child's paediatrician records and used to calculate BMI. BMI was calculated using the following formula: BMI = weight (kg)/height (m)^2^.

#### 2.2.2 Perceived overeating

Repeated measures of mother‐reported child overeating were assessed at four timepoints: at the age of 3, 5, 7 and 11 years. Mothers were asked if their child had overeaten in the last year and whether they were worried about their child overeating. Mothers were given the following response options: ‘no/did not happen’, ‘not worried’, ‘a bit worried’ and ‘greatly worried’. The top two categories (‘a bit worried’ and ‘greatly worried’) were combined to avoid very low frequencies, resulting in a 3‐point scale of perceived overeating. This item reflects the maternal perception of their child's overeating, the same measure has been used in previous studies.^22^, ^23^, ^24^

#### 2.2.3 Maternal BMI before pregnancy

Height and weight (before pregnancy) were self‐reported by mothers. The questionnaire was administered during pregnancy. BMI was calculated using the following formula: BMI = weight (kg)/height (m)^2^.

#### 2.2.4 Adverse Childhood Experiences

The information on ACEs was collected from mothers/primary caregivers, fathers/parents and paediatricians at 6 and 18 months of age. Since the ELSPAC study is a sister study of the British ALSPAC study, ELSPAC questionnaires were created by translating the original ALSPAC questionnaires from English to Czech language, and thus the items assessing the childhood adversities corresponded to the original ALSPAC items. We assessed the types of ACEs and the overall ACE score in accordance with the previously developed guidelines for ALSPAC^25^ and previous studies.^26^, ^27^ For the current study, we defined ACEs as eight intra‐familial adversities^28^ the child might have been exposed to up to 18 months of age. The overview of ACEs is provided in Table 1. Each ACE was coded as a binary variable (1 indicating the presence and 0 indicating the absence of a specific ACE during the period). The presented ACEs were summed into a score ranging from 0 to 8 and subsequently recoded into five categories: 0, 1, 2, 3 and ≥4 ACEs.

**TABLE 1:** The descriptions of the assessed ACEs.

Sexual abuse	Mother responded affirmatively to the item ‘Your child was sexually abused’.
Physical abuse	Mother, father or both responded affirmatively to any of the following items: (a) ‘You were physically cruel to your children’ and (b) ‘Your partner was physically cruel to your children’.
Emotional abuse	Mother, father or both responded affirmatively to any of the following items: (a) ‘You were emotionally cruel to your children’ and (b) ‘Your partner was emotionally cruel to your children’.
Parental mental illness	Mother, father or both: (a) scored 13 or more on the Edinburgh Postnatal Depression Scale, indicating an increased risk of depression disorder, (b) reported a suicide attempt or (c) reported own or partner's doctor consultations for depression or anxiety.
Parental offending	Mother, father or both responded affirmatively to any of the following items: (a) ‘You were in trouble with the law’; (b) ‘Your partner was in trouble with the law’ and (c) ‘You were convicted of an offence’.
Parental divorce or separation	Mother, father or both: (a) reported a change in the marital status from married at an earlier time point to divorced/separated at a later time point or answered affirmatively any of the following questions: (b) ‘You got divorced’; (c) ‘Your partner left you’ and (d) ‘You broke up with your partner’.
Parental conflict and violence	Mother, father or both responded affirmatively to any of the following items: (a) ‘Your partner was physically cruel to you’ or (b) ‘Your partner was emotionally cruel to you’.
Parental substance use	Mother, father or both reported: (a) daily alcohol consumption of 3 or more drinks; (b) own or partner's doctor consultations for problems with alcohol; (c) own or partner's daily marijuana use or (d) any use of heroin, cocaine or crack.

#### 2.2.5 Maternal education

The highest attained education of the mother was assessed based on the questionnaire administered during pregnancy. Educational attainment was classified into three groups: ‘high’, including mothers with higher professional or university education; ‘middle’, defined as high school education and ‘low’, defined as elementary or vocational education without a final graduation exam.

#### 2.2.6 Single‐parent household

Single parenthood was assessed by a questionnaire administered to the mother at 18 months of the children. Mothers who reported being without a partner or not having a partner living in the same home with them were coded as living in a single‐parent household.

#### 2.2.7 Financial difficulties

Financial difficulties were assessed by five questions administered to the mother at 18 months of the children. Mothers used a four‐point Likert scale to answer how difficult it is to secure the family with the following five things: food, clothes, heating, rent/other fees and things necessary for the child.

### 2.3 Data analysis

Continuous variables were described using means, whilst categorical variables were described using percentages. We modelled the longitudinal associations between BMI and perceived overeating using the random intercept cross‐lagged panel model (RI‐CLPM) to answer our research questions.^29^, ^30^ The benefit of this approach compared to a more common cross‐lagged panel model lies in its ability to decompose the variance into the within‐ and between‐person effects, which enables the researchers to estimate the stable individual differences (between‐person) as well as effects from one timepoint to the other (within‐person). We estimated the autoregressive paths for the four timepoints of BMI and four timepoints of perceived overeating (3, 5, 7 and 11 years), reflecting the within‐individual stability in the same construct across time. Then, we estimated the cross‐lagged effects between these two constructs, reflecting the time‐lagged effect of one trait on the other within individuals. Random intercepts of BMI and perceived overeating, represented as latent factors with the four BMI or perceived overeating timepoints as indicators, were assessed, and these two random intercepts were allowed to covary. These latent factors represent stable, trait‐like components of BMI and perceived overeating, indicating the individual's rank order on these variables compared to the sample (for more details on the method, please see Mulder et al. 2020^30^ and Hamaker et al. 2015^29^). The two random intercepts were regressed on the baseline variables. We included factors that indicate socioeconomic disadvantage (financial difficulties, low maternal education and single parenting), negative early childhood experiences (ACEs) and maternal BMI, reflecting both the genetic and environmental transgenerational effects on a child's BMI and perceived overeating. All factors were assessed at previous timepoints from our dependent variables. According to the literature, the prevalence of ACEs is higher in families with socioeconomic disadvantage.^31^, ^32^ In accordance with that notion, the ACEs were regressed on maternal education, financial difficulties and single parenting to assess the potential mediating role of ACEs in the association between socioeconomic disadvantage and the longitudinal development of BMI and perceived overeating. Financial difficulties were modelled as a latent factor with five indicators (see Supplementary Figure 1 for results from the financial difficulties CFA model). The significance of mediation was estimated using the delta method. The estimates from cross‐lagged paths were compared using the Wald test. Descriptive statistics were performed using STATA^33^ software (version 16.0, StataCorp, College Station, TX, USA). An adequate model fit was defined as CFI ≥0.90 and RMSEA <0.08.^34^ The full structural model (RI‐CLPM) was estimated in Mplus 8.10.^35^ We used the robust weighted least squares (WLSMV) estimator for the structural model.

### 2.4 Handling missing data

The percentage of missing data across all study variables ranged from 0.03% to 56.60% (Table 2). Given the amount of missing data in our sample, we have utilized multiple imputations in Mplus based on Markov Chain Monte Carlo simulation to handle missing data. Multiple imputations are preferred in cases where the amount of missing data is non‐trivial, and the pattern of missingness is not completely random.^36^ The imputation model included all study variables along with birth weight, paternal education and paternal BMI as auxiliary variables. One hundred imputed data sets were created, and the presented results from structural models are pooled estimates using Rubin's method.^37^

**TABLE 2:** Descriptives.

Variable	Mean (SD)/%	% missing	Variable	Mean (SD)/%	% missing
Females	48.43	0.00	Maternal education		23.30
BMI (kg/m^2^)			Primary	38.70	
3 years	15.59 (1.59)	15.36	Secondary	43.02	
5 years	15.33 (1.69)	24.46	Tertiary	18.28	
7 years	15.68 (1.96)	28.93	Financial difficulties		
11 years	17.86 (3.01)	39.29	Difficulties to pay for food		33.78
Perceived overeating—3 years		33.72	No difficulties	62.77	
No	88.11		Slightly difficult	22.63	
Yes, not worried	6.06		Fairly difficult	10.58	
Yes, and worried	5.83		Very difficult	4.02	
Perceived overeating—5 years		34.34	Difficulties to pay for clothes		33.76
No	85.75		No difficulties	33.91	
Yes, not worried	7.72		Slightly difficult	34.35	
Yes, and worried	6.53		Fairly difficult	21.19	
Perceived overeating—7 years		40.17	Very difficult	10.55	
No	84.56		Difficulties to pay for heating		34.23
Yes, not worried	8.01		No difficulties	70.78	
Yes, and worried	7.43		Slightly difficult	17.03	
Perceived overeating—11 years		53.45	Fairly difficult	8.56	
No	83.11		Very difficult	3.63	
Yes, not worried	7.84		Difficulties to pay for rent or loans		34.13
Yes, and worried	9.05		No difficulties	55.05	
Maternal BMI (kg/m^2^)	22.05 (3.30)	27.00	Slightly difficult	26.41	
ACEs		34.91	Fairly difficult	12.38	
0	53.68		Very difficult	6.16	
1	24.84		Difficulties to pay for necessary things for children		33.90
2	10.56		No difficulties	49.34	
3	6.14		Slightly difficult	31.42	
≥4	4.77		Fairly difficult	13.83	
Single parent family	6.30	33.45	Very difficult	5.40	

*Note*: Results are reported as percentage or mean (standard deviation).

### 2.5 Ethical statements

Informed consent was obtained from all participants. The study was approved by the ELSPAC Law and Ethics Committee and local research ethics committees. The secondary use of all ELSPAC study data was approved by the (C)ELSPAC Ethics Committee (Ref. No. ELSPAC/EK/1/2014, date 09/17/2014).

## 3 RESULTS

### 3.1 Subjects characteristics

The descriptive statistics of study variables from the non‐imputed data are shown in Table 2. There were slightly more males in the sample (51.57%). The mean maternal BMI was 22.05. About 38.70% of mothers had less than high school as their highest attained education, 43.02% of mothers were high school graduates and 18.28% had college degrees. About 6.3% of mothers lived in single‐parent households. The average child's BMI was stable between the ages of 3, 5 and 7 (15.59, 15.33 and 15.68 kg/m^2^) and slightly increased at the age of 11 (17.86 kg/m^2^). The percentage of children with perceived overeating increased with age, from about 11.89% at age 3 to 14.25% at age 5, 15.44% at age 7 and 16.89% at age 11. About 53.68% of mothers reported no adverse childhood experience by the first 18 months of the child's age, 24.84% reported 1 ACE, 10.56% reported 2 ACEs, 6.14% reported 3 ACEs and 4.77% reported 4 ACEs or more. For descriptive statistics of imputed sample see Table S1.

### 3.2 Estimated within and between‐person effects in the RI‐CLPM

**FIGURE 1:**
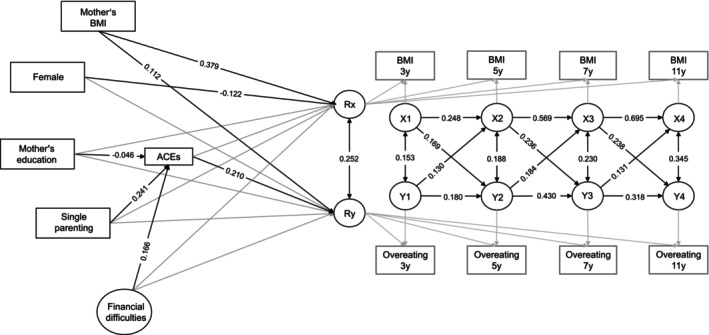
Standardized coefficients of RI‐CLPM. Reported results are significant at *p* < 0.05. Rx reflects the random intercept of BMI across the four timepoints; Ry reflects the random intercept of perceived overeating across the four timepoints; X1–X4 are latent factors representing the within‐person variance in BMI in each timepoint; Y1–Y4 are latent factors representing the within‐person variance in perceived overeating in each timepoint.

The standardized results from the structural model are shown in Table 3 and Figure 1. The model showed an adequate fit, *χ* ^2^(105) = 853.480, *p* < 0.001, CFI = 0.985, RMSEA = 0.037. The correlation between latent intercepts of perceived overeating and BMI was *r* = 0.252, *p* < 0.001, showing that children with higher levels of BMI across the four waves were also found to be more likely to overeat.

**TABLE 3:** Estimated within and between‐person effects in the RI‐CLPM.

Predictor		Outcome	*β*	95% CI	*p*	Predictor		Outcome	*β*	95% CI	*p*
Within‐person effects						Between‐person effects					
Auto‐regressive paths						Predictors on BMI latent intercept					
BMI 3y	→	BMI 5y	0.248	0.090; 0.406	0.002	Female	→	Rx	−0.122	−0.167; −0.077	<0.001
BMI 5y	→	BMI 7y	0.569	0.505; 0.633	<0.001	Maternal BMI	→	Rx	0.379	0.305; 0.452	<0.001
BMI 7y	→	BMI 11y	0.695	0.661; 0.730	<0.001	Maternal education	→	Rx	−0.046	−0.095; 0.003	0.064
Overeating^a^ 3y	→	Overeating^a^ 5y	0.180	0.038; 0.322	0.013	Financial difficulties	→	Rx	0.000	−0.059; 0.059	0.993
Overeating^a^ 5y	→	Overeating^a^ 7y	0.430	0.327; 0.534	<0.001	Single parenting	→	Rx	0.024	−0.031; 0.079	0.387
Overeating^a^ 7y	→	Overeating^a^ 11y	0.318	0.182; 0.454	<0.001	ACEs	→	Rx	−0.013	−0.073; 0.048	0.678
Cross lagged paths						Predictors on overeating^a^ latent intercept					
BMI 3y	→	Overeating^a^ 5y	0.169	0.056; 0.282	0.003	Female	→	Ry	−0.010	−0.070; 0.049	0.732
BMI 5y	→	Overeating^a^ 7y	0.236	0.158; 0.314	<0.001	Maternal BMI	→	Ry	0.112	0.049; 0.175	<0.001
BMI 7y	→	Overeating^a^ 11y	0.238	0.144; 0.333	<0.001	Maternal education	→	Ry	0.053	−0.013; 0.118	0.116
Overeating^a^ 3y	→	BMI 5y	0.130	0.012; 0.248	0.030	Financial difficulties	→	Ry	0.068	−0.005; 0.141	0.067
Overeating^a^ 5y	→	BMI 7y	0.184	0.126; 0.241	<0.001	Single parenting	→	Ry	−0.045	−0.116; 0.027	0.219
Overeating^a^ 7y	→	BMI 11y	0.131	0.075; 0.187	<0.001	ACEs	→	Ry	0.210	0.130; 0.291	<0.001
Residual correlations						Social predictors on ACEs					
BMI 3y	With	Overeating^a^ 3y	0.153	0.021; 0.285	0.023	Maternal education	→	ACEs	−0.046	−0.086; −0.006	0.025
BMI 5y	With	Overeating^a^ 5y	0.188	0.098; 0.277	<0.001	Financial difficulties	→	ACEs	0.166	0.126; 0.205	<0.001
BMI 7y	With	Overeating^a^ 7y	0.230	0.162; 0.299	<0.001	Single parenting	→	ACEs	0.241	0.207; 0.276	<0.001
BMI 11y	With	Overeating^a^ 11y	0.345	0.271; 0.419	<0.001	Correlation of latent intercepts					
						Rx	With	Ry	0.252	0.081; 0.423	0.004

*Note*: Model fit—*χ* ^2^(107) =853.480 CFI = 0.985, RMSEA = 0.037. Rx: BMI latent intercept. Ry: Overeating latent intercept. *β* represents the standardized coefficients.

^a^ Overeating represents parents‐reported overeating.

The time‐varying portion of the model showed temporal stability in the development of perceived overeating and substantial stability in the BMI trajectory (all autoregressive paths statistically significant at *p* < 0.05). The cross‐sectional effects showed positive correlations between individual BMI and perceived overeating in each wave. This association linearly increased with time, age 3: *r* = 0.153, age 5: *r* = 0.188, age 7: *r* = 0.230, age 11: *r* = 0.345, all *p* < 0.05. The cross‐lagged paths showed a consistent association between BMI and later overeating (*β* = 0.169; *β* = 0.236; *β* = 0.238; all *p* < 0.05). Similarly, significant findings were also found for the association between perceived overeating and BMI (*β* = 0.130; *β* = 0.184; *β* = 0.131; all *p* < 0.05). Wald test showed no significant differences in cross‐lagged effects from age 3 to age 5: W (1) = 0.12, *p* = 0.718; age 5 to age 7: W (1) = 1.52, *p* = 0.217 and age 7 to age 11: W (1) = 3.12, *p* = 0.077.

Maternal BMI was found to be significantly associated with both the child's BMI (*β* = 0.379, *p* < 0.001) and perceived overeating (*β* = 0.112, *p* < 0.001). Girls were found to have lower levels of BMI (*β* = −0.122, *p* < 0.001), yet no sex differences were found for overeating. Neither maternal education, single‐parent family nor financial difficulties were significantly associated with BMI or overeating. The cumulative sum of ACEs was significantly associated with perceived overeating (*β* = 0.210, *p* < 0.001), but it was not associated with BMI. ACEs were negatively associated with maternal education (*β* = −0.046, *p* = 0.025), whilst they were positively associated with single parenting (*β* = 0.241, *p* < 0.001), as well as financial difficulties (*β* = 0.166, *p* < 0.001). All the factors accounted for 17% of the variance in BMI and 7% of the variance in perceived overeating.

Turning to indirect effects, no significant indirect effects were found for child's BMI. On the other hand, ACEs were found to mediate the effect of maternal education on perceived overeating, *β* = −0.010, *p* = 0.039. Positive indirect effect through ACEs was also found for financial difficulties (*β* = 0.035, *p* < 0.001), and single parenthood (*β* = 0.051, *p* < 0.001) (Table 4).

**TABLE 4:** Standardized total and indirect effects of the associations between predictors and latent intercepts mediated by ACEs.

	Total effect			Indirect effect		
	*β*	95% CI	*p*	*β*	95% CI	*p*
To BMI						
Maternal education	−0.046	−0.095; 0.003	0.067	0.001	−0.002; 0.004	0.701
Financial difficulties	−0.002	−0.060; 0.055	0.936	−0.002	−0.012; 0.008	0.680
Single parenting	0.021	−0.031; 0.074	0.430	−0.003	−0.018; 0.012	0.675
To perceived overeating						
Maternal education	0.043	−0.023; 0.109	0.202	**−0.010**	**−0.019; 0.000**	**0.039**
Financial difficulties	**0.103**	**0.031; 0.175**	**0.005**	**0.035**	**0.019; 0.050**	**<0.001**
Single parenting	0.006	−0.062; 0.074	0.860	**0.051**	**0.030; 0.071**	**<0.001**

*Note*: Bold values represent results significant at *p* < 0.05 level.

## 4 DISCUSSION

The current study aimed to 1) longitudinally investigate the directionality and dynamics of the association between BMI and perceived overeating and 2) assess the longitudinal effects of early childhood factors on the co‐development of perceived overeating and BMI throughout childhood. The results indicated an increase in BMI and perceived overeating over time. The effect of BMI on perceived overeating was generally higher than that of perceived overeating on BMI, but all the bidirectional cross‐legged effects were not statistically different. The observed effects of early life factors on BMI and perceived overeating potentially suggest two distinct pathways of childhood obesity development.

The first pathway reflects an increase in BMI starting in early childhood, followed by a higher likelihood of perceived overeating. These results replicated the previous evidence from other studies.^19^, ^20^, ^38^ This direction might seem counter‐intuitive, yet it is clearly supported by previous literature. The obesity‐associated gene polymorphism and excessive adipose tissue in the body have been associated with binge eating and overeating in later childhood and adolescence.^38^ The probable underlying mechanism for this association revolves around the up‐regulation of appetite and reduced satiety responsiveness.^19^, ^39^ The second pathway involves continuous perceived overeating beginning in early childhood, leading to a gradual BMI increase. Overeating can be understood as excess food consumption, which is usually related to increased energy intake. The long‐term energy imbalance when energy intake exceeds energy expenditure leads to the accumulation of adiposity tissue and connected weight gain.^19^, ^20^, ^40^ Our results also suggested a potential increase in the strength of the effect in auto‐regressive and cross‐lagged paths after the fifth year of children's age, indicating the deepening long‐term consequences of early life conditions. The timing of the increase after the fifth year of age may possibly be related to a critical period called adiposity rebound, which refers to an increase in BMI after its sharp drop in early childhood.^41^ After the adiposity rebound, the BMI trends tend to be more stable than in previous years of life.^42^, ^43^

Conceptually, the critical periods of the life course refer to specific time windows during an individual's life when certain exposures or experiences can have a significant and enduring influence on health outcomes. Whilst the immediate consequences may not always be apparent, they can set the stage for a cascade of long‐term health outcomes.^3^ For instance, early life exposure to social disadvantage has been found to significantly increase the risk of obesity in adulthood.^44^, ^45^ Similarly, specific health conditions such as increased BMI during childhood critical periods,^46^ markedly elevate the likelihood of developing obesity in adulthood.^43^, ^46^, ^47^ The current study investigated the effect of early life social predictors and maternal BMI on longitudinal trends in both child's BMI and a perceived child's overeating. A child's BMI was associated with maternal BMI, suggesting a strong role of inherited obesity. In this context, the term inherited obesity can be understood as genetic inheritance, together with the potential adoption of the parent's behaviours.^48^ Although the genetic predisposition itself is an unmodifiable factor, parental and family behaviours can be subject to intervention. The effectiveness of individual lifestyle intervention for childhood obesity has been proven before,^49^ yet these interventions are usually applied at a moment when unhealthy weight or obesity is already developed and therefore may have limited effect on the complex origin of childhood obesity.^49^ The interventions focused on preventing the occurrence of childhood obesity are often related to providing information about nutrition, physical activity and a healthy lifestyle, typically conducted in schools or other community facilities,^50^ and their effectiveness has been proved by previous evidence.^51^ Nevertheless, despite the implementation of various interventions and strategies, the prevalence of childhood obesity continues to increase. A common thread amongst these efforts is their focus on modifying individual behaviours, whether those of parents or children. Consequently, they may fail to adequately consider the complexity of childhood obesity predictors and address the broader upstream determinants over the life course that may underlie these individual behaviours.

In the current study, we observed a significant effect of upstream social determinants in the second pathway of obesity development, based on perceived overeating followed by an increase in BMI. Although higher levels of perceived overeating were also associated with maternal BMI, a stronger effect was found for ACEs. Previous studies described the effect of ACEs on obesity in childhood^8^, ^14^, ^15^ but also the long‐term impacts on obesity in adulthood.^15^ The underlying mechanism could be partially attributed to physiological stress regulation. ACEs may induce stress, triggering homeostatic adjustments and disruptions in the hypothalamic–pituitary–adrenal axis. This axis plays a pivotal role in regulating the secretion and function of hormones linked to appetite, such as cortisol, ghrelin, leptin and insulin.^52^ However, it is important to highlight that ACEs themselves are affected by social factors and could, therefore, contribute to explaining the causes of social inequalities in childhood obesity.^31^, ^32^, ^53^ The novelty of our study includes the investigation of a broader context. Our results indicated that ACEs were positively associated with low maternal education, financial difficulties and single parenting, and as such, served as a mediator between these social factors and the risk for perceived overeating.^54^ Disadvantaged families often face increased levels of stress stemming from material deprivation, structural inequalities or stigmatization.^54^ Material deprivation is closely related to food insecurity, which has been previously found to be associated with inappropriate dietary patterns in children, such as low consumption of vegetables and whole grains^55^ and increased consumption of added sugar and ultra‐processed food.^56^ However, the impact of food insecurity exists also beyond the nutritional concerns; it is an important stressor affecting child development and mental health.^54^ Besides that, living in inappropriate life conditions affects psychological susceptibility, risk for substance abuse and overall mental health. Children living in disadvantaged social groups have oftentimes been found to be exposed to higher levels of stress resulting from exposure to ACEs.^32^ Such children may resort to overeating as a means to self‐soothe or cope with negative emotions. This behaviour is commonly referred to as emotional overeating.^57^, ^58^

The mediation role of ACEs in social inequalities in childhood obesity was previously investigated by Priest et al.^59^ in a sample of 1873 children from the LSAC cohort and 7085 children from the ALSPAC cohort, who showed that the exposure to multiple ACEs significantly mediated the association between low maternal education and increased BMI in children.^59^ Similar observations were reported in the UK Millennium Cohort Study, where ACEs explained about 19% of the effect of maternal education on adolescent adiposity.^53^ The inequalities in the prevalence of childhood obesity thus most likely arise from the accumulation and chaining of social stressors, the effect of which is mediated by ACEs.

This cumulative risk amongst families with low SES makes them the primary target of tailored efforts to prevent or curb childhood obesity. The findings of this study emphasize the need for public health initiatives to focus on a broader spectrum of determinants, which may involve investment in support systems that consider the unique stressors faced by families with socioeconomic disadvantage.

### 4.1 Strengths and limitations

The current study has several strengths worth mentioning. One of the study's major strengths is the utilization of a large sample of mothers and their children, including more than 5000 individuals at the baseline. Furthermore, this sample was followed through an extensive time period, spanning from infancy to late childhood. The large sample size and the long timespan covered in the analyses allow for more robust conclusions regarding childhood obesity development. The validity of the present findings is further bolstered by the comprehensive analytical approach employing a random intercept cross‐lagged model, which enabled us to model the bivariate relationships between BMI and perceived overeating over time at the within‐person level whilst simultaneously modelling the effect of early childhood predictors on overall levels of these outcomes.

There are also several limitations of the study. There was a substantial sample attrition at the later timepoints. We used multiple imputations to deal with missing data, but the high level of attrition may introduce potential bias into the findings, as the characteristics of those who dropped out may differ from those who remained in the study. Furthermore, child overeating was mother‐reported and, therefore, is subject to reporting bias. The wording of the response options reflected mother's perception of their child's overeating and whether it worried them. As such, this item reflects the maternal perception of their child's overeating and might be affected by maternal characteristics (i.e., how mothers differ in what amount of food consumed they consider overeating and what amount worries them). As children grow up and begin to have more meals outside the home, parents are increasingly less likely to monitor their eating behaviours. However, parental reports remain the most commonly used measure of child eating behaviours, as young children cannot reliably report their own behaviour. Such limitation is therefore inherent to questionnaire‐based studies, and the same measure of overeating has been used in other studies.^22^, ^60^ Moreover, maternal BMI before pregnancy was estimated based on weight reported by mothers retrospectively and may, therefore, be imprecise. Finally, mothers in the sample came from middle and large‐sized towns. Although there have been some changes in residential addresses throughout the course of the ELSPAC data collection, the sample is reflective mainly of the urban population; therefore, results might not be extrapolated to rural populations.

## 5 CONCLUSION

This study demonstrated the bidirectional longitudinal association between a child's BMI and perceived overeating. The analysis of early childhood factors suggested two potential pathways of childhood obesity development. The first pathway was affected mainly by maternal BMI and reflected in increased BMI starting in early childhood, followed by a higher likelihood of perceived overeating. The second pathway was affected by ACEs and reflected in continuous perceived overeating beginning in early childhood, leading to a gradual increase in BMI. Additionally, ACEs acted as a mediator of the effect of social factors and therefore potentially contribute to the development of social inequalities in childhood obesity. These findings demonstrate that the roots of childhood obesity extend beyond immediate individual factors. Therefore, to reduce the burden of childhood obesity, public health interventions must extend their scope to early life upstream determinants and sufficiently address the complex origin of childhood obesity.

## AUTHOR CONTRIBUTIONS

AB, AK and HP conceived and designed the study. AB and AK analysed the data. AB and AK wrote the first draft of the manuscript with the support of GK and MB. HP, JK and MB provided critical revisions. All authors read and approved the submitted manuscript.

## FUNDING INFORMATION

This work has received funding from the European Union's Horizon 2020 research and innovation programme under grant agreement No. 857487 (R‐Exposome Chair) and was supported under grant agreement No. 857560 (CETOCOEN Excellence). This publication reflects only the author's view, and the European Commission is not responsible for any use that may be made of the information it contains. Authors thank the RECETOX Research Infrastructure (No. LM2023069) financed by the Ministry of Education, Youth and Sports, and the Operational Programme Research, Development and Education (the CETOCOEN EXCELLENCE project No. CZ.02.1.01/0.0/0.0/17_043/0009632) for supportive background. This output was supported by the National Institute for Research of Metabolic and Cardiovascular Diseases (Programme EXCELES, ID Project No. LX22NPO5104)—Funded by the European Union—Next Generation EU.

## CONFLICT OF INTEREST STATEMENT

No conflict of interest was declared.

## Supporting information


**Supplementary Table S1.** Descriptives of imputed datasets.
**Supplementary Figure S1.** The latent variable representing financial difficulties. All reported standardized coefficients significant at *p* < 0.001.

## Data Availability

The data are available upon request and with permission of RECETOX, Faculty of Science, Masaryk University.
