# Supplementary material for: Early life social conditions and adverse experiences are associated with childhood BMI and perceived overeating
Source: Pediatr Obes. 2024 Oct 8;20(4):e13179. doi: 10.1111/ijpo.13179 (PMC11936712; doi:10.1111/ijpo.13179)

Early life social conditions and adverse experiences are associated with childhood BMI and perceived overeating

Anna Bartoskova Polcrova[1], Gabriela Ksinan Jiskrova[1], Martin Bobak[1,2], Hynek Pikhart[1,2], Jana Klánová[1], Albert J. Ksinan[1]

[1]RECETOX, Faculty of Science, Masaryk University, Kotlarska 2, Brno, Czech Republic

[2]Department of Epidemiology and Public Health, University College London, United Kingdom

Corresponding author:

Albert J. Ksinan, RECETOX, Faculty of Science, Masaryk University, Kotlarska 2, Brno, Czech Republic

Email: albert.ksinan@recetox.muni.cz

Supplementary table 1: Descriptives of imputed datasets

Variable	Mean (SD) / %	Variable	Mean (SD) / %
**Females**	48.43	**Maternal education**	
**BMI (kg/m²)**		Primary	38.6
3 years	15.58	Secondary	43.0
5 years	15.32	Tertiary	18.4
7 years	15.70	**Financial difficulties**	
11 years	17.87	**Difficulties to pay for food**	
Perceived **overeating - 3 years**		No difficulties	60.0
No	88.0	Slightly difficult	25.8
Yes, not worried	6.1	Fairly difficult	10.9
Yes, and worried	5.9	Very difficult	3.3
Perceived **overeating - 5 years**		**Difficulties to pay for clothes**	
No	85.8	No difficulties	32.2
Yes, not worried	7.7	Slightly difficult	34.3
Yes, and worried	6.5	Fairly difficult	23.6
Perceived **overeating - 7 years**		Very difficult	9.8
No	84.6	**Difficulties to pay for heating**	
Yes, not worried	8.0	No difficulties	67.8
Yes, and worried	7.5	Slightly difficult	20.3
Perceived **overeating - 11 years**		Fairly difficult	9.1
No	82.5	Very difficult	2.9
Yes, not worried	8.0	**Difficulties to pay for rent or loans**	
Yes, and worried	9.5	No difficulties	51.9
**Maternal BMI (kg/m²)**	22.1	Slightly difficult	29.0
**ACEs**		Fairly difficult	13.8
0	53.3	Very difficult	5.3
1	24.9	**Difficulties to pay for necessary things for children**	
2	10.7	No difficulties	46.9
3	6.3	Slightly difficult	33.6
≥4	4.9	Fairly difficult	14.9
**Single parent family**	6.6	Very difficult	4.6

Results are reported as percentage or mean (standard deviation).

Supplementary figure 1: The latent variable representing financial difficulties. All reported standardized coefficients significant at $p<0.001$.

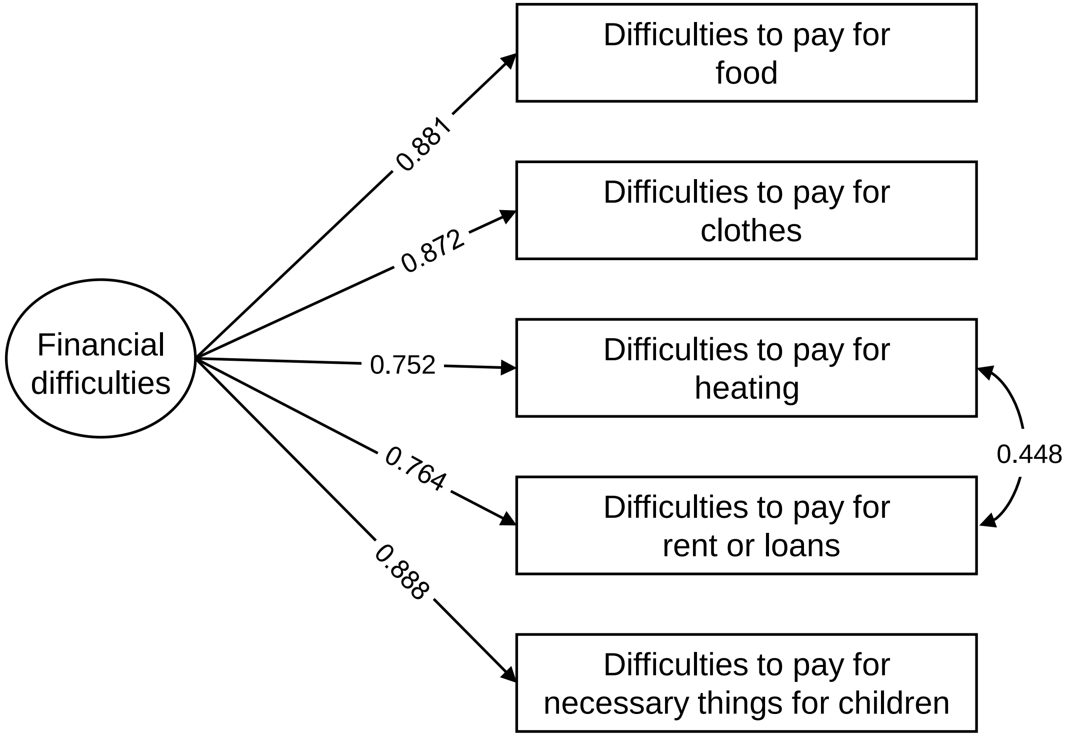

Supplement: Supplementary file 1 — Supplementary Table S1. Descriptives of imputed datasets. Supplementary Figure S1. The latent variable representing financial difficulties. All reported standardized coefficients significant at p < 0.001. [file IJPO-20-e13179-s001.pdf]
